# Genome mining, *in silico* validation and phase selection of a novel aldo-keto reductase from *Candida glabrata* for biotransformation

**DOI:** 10.1080/21655979.2017.1342911

**Published:** 2017-07-13

**Authors:** Souvik Basak, Nanda Gopal Sahoo, Angayar K. Pavanasam

**Affiliations:** a Dr. B.C. Roy College of Pharmacy & Allied Health Sciences, Durgapur, WB, India; b Nanoscience and Nanotechnology Centre, Department of Chemistry, Kumaun University, Nainital, Uttarakhand, India; c International College of Engineering and Management (University of Central Lancashire, UK Affiliation), Muscat, Oman

**Keywords:** binding site, cofactor regeneration, docking, DNA shuffling, homology match

## Abstract

Previously, we published cloning, overexpression, characterization and subsequent exploitation of a carbonyl reductase (*cr*) gene, belonging to general family aldo-keto reductase from *Candida glabrata* CBS138 to convert keto ester (COBE) to a chiral alcohol (ethyl-4-chloro-3-hydroxybutanoate or CHBE). Exploiting global transcription factor CRP, rDNA and transporter engineering, we have improved batch production of CHBE by trinomial bioengineering. Herein, we present the exploration of *cr* gene in *Candida glabrata* CBS138 through genome mining approach, *in silico* validation of its activity and selection of its biocatalytic phase. For exploration of the gene under investigation, 3 template genes were chosen namely *Saccharomyces cerevisae* YDR541c, YGL157w and YOL151w. The CR showed significant homology match, overlapping of substrate binding site and NADPH binding site with the template proteins. The binding affinity of COBE toward CR (−4.6 Kcal/ mol) was found higher than that of the template proteins (−3.5 to −4.5 Kcal/ mol). Biphasic biocatalysis with cofactor regeneration improved product titer 4∼5 times better than monophasic biotransformation. Currently we are working on DNA Shuffling as a next level of strain engineering and we demonstrate this approach herein as a future strategy of biochemical engineering.

## Introduction

In previous studies, many enzymes of Aldo-keto Reductase (AKR) and Carbonyl reductase (CR) family were cloned, characterized and used in the asymmetric synthesis of (*S*)-CHBE^1-4^ and (*R*) CHBE.^5^ AKR & CR find extensive applications in pharmaceutical industry. For example, they are used as key chiral intermediates in the enantioselective synthesis of slagenins B and C, they serve as 2 potential compounds against murine leukemia, they are also used for synthesis of HMG-CoA reductase inhibitors (hypolipidemic agents) and can be converted into 1,4 dihydropyridine type blocker (antihypertensive agents).^1-5^ Similarly, they are also used for conversion of ethyl-4-chloro-3-oxobutanoate (COBE) to optically active ethyl-4-chloro-3-hydroxybutanoate (CHBE) as CHBE serve as a versatile precursor for pharmcalogically valuable products.^1-5^ Although attempts have been made to augment bioconversion by either genetically manipulating the biocatalytic system with cofactor regeneration or fabricating the reaction media with single or multiple solvents, productivity has often faced shortfall due to obtaining higher reaction rate only at small substrate concentration (5∼230 mM) thus eliciting a limiting batch output within the reactor.^6-9^ Hence, establishment of a biocatalysis system with industrial competence has always been a prime search through the years which has driven researchers to find out newer proteins with improved activity and higher productivity.

In this context, in our previous publication,^10^ we have reported the cloning, expression and purification of a carbonyl reductase from *Candida glabrata* CBS 138. We have also introduced the recombinant gene into globally engineered strain constructed by manipulating global transcription factor CRP. The improved tolerance by the host cell against organic phase, which had been added as an essential component of the biotransformation, led the output of the process shoot outstandingly higher. However, detailed methodology of the gene exploration together with optimization of the biocatalysis medium had not been described in details in the previous report. Thus, exploration of the gene through bioinformatics guided approach and its validation through docking studies has been presented in this work. In addition, this work also focuses on enhancing cell phenotype through DNA shuffling. Preliminary data obtained from DNA shuffling appears to be promising with better product titer and hence it is envisaged to extend our research on improvement of microbial cell factory through the above mentioned techniques.

### Discovery of CR protein from *Candida glabrata* CBS138

**Figure 1.**
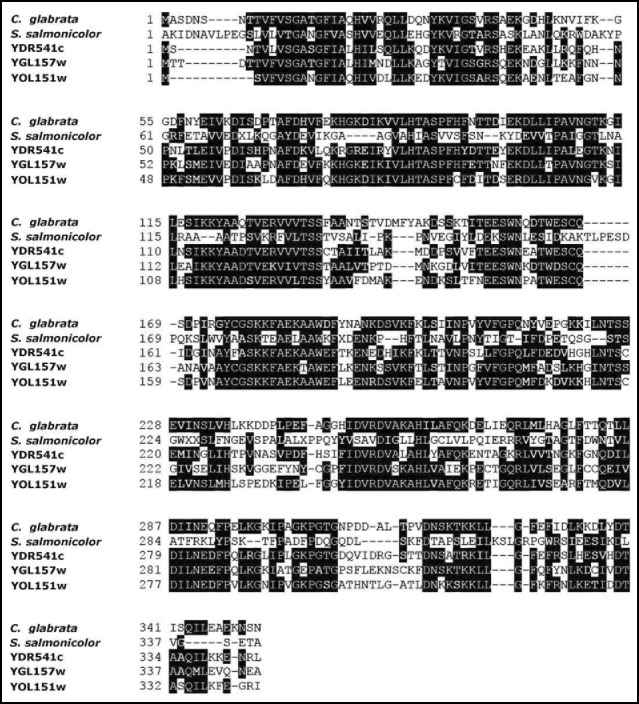
Multiple sequence alignment of XP_445913.1 from *Candida glabrata* CBS138 with other reported Aldo-keto Reductase group of proteins namely one aldose reductase from *Sporobolomyces salmonicolor* and 3 *Saccharomyces cerevisiae* derived genes encoding carbonyl reductases (YDR541c, YGL157w, YOL51w). The conserved domains are highlighted as black and gray boxes

Three open reading frames namely *S. cerevisiae* YDR541c, YGL157w and YOL151w, reported for encoding aldo-keto reductases,^11^ were subjected to Protein BLAST (BLASTP) along with 2 crystallographically elucidated proteins of the same class namely aldehyde reductase 2 from *Sporobolomyces salmonicolor* AKU4429 (PDB ID: 1UJM) and an aldehyde reductase from *Sporidiobolus salmonicolor* (PDB ID: 1Y1P). The BLASTP results from all attempts revealed the repetitive hypothetical protein from *Candida glabrata* CBS138 (Protein ID: XP_445913.1). The BLAST scores revealed that the yeast proteins bear a plausible 55% to 62% identity to the target protein together with a staggering 98% sequence coverage with the latter. In contrast, 1UJM and 1Y1P possessed only 30% identity with the CR protein, however their 95% sequence swap with the target protein led us to a reliable approximation that XP_445913.1 from *Candida glabrata* CBS138 might belong to the same family as *S. cerevisiae* YDR541c, YGL157w, YOL151w together with 1UJM and 1Y1P. Multiple sequence alignment of the structures showed that they preserve a high degree of homology match with each other (Fig. 1) with a comprehensive amount of conserved amino acids at most of the positions.

## Homology modeling

For further investigations of XP_445913.1 from *C. glabrata* CBS138, homology model of the same was constructed using 5 template proteins as used for BLAST. In this process, primary homology models have been created for *S. cerevisiae* YDR541c, YGL157w and YOL151w since crystallographic structure has not been elucidated till date for these proteins. Homology modeling of all the 4 proteins were acquiesced using MODELLER9.12 (www.scilab.org/modeler). The overall folding of the homology model structure was same (RMSD 0.466) as analyzed by swiss pdb.

**Figure 2.**
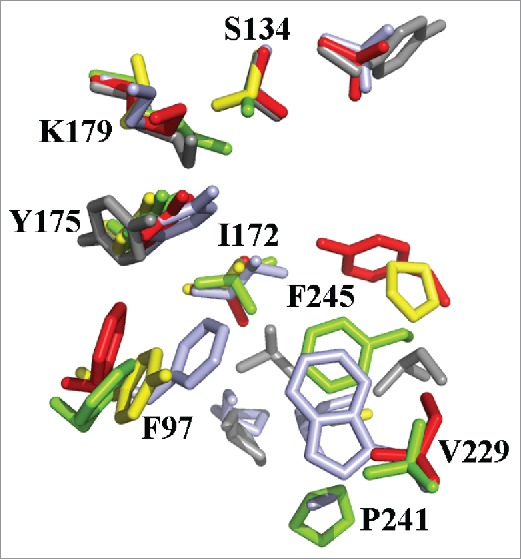
Positions of important amino acids for COBE binding shown in stick representation by pyMOL. Overlap of crystal structure from *S. Salmonicolor* (light blue) and homology model structure from *C. glabrata* (green), *S. cerevisae* YDR541C (yellow), *S. cerevisae* YGL157W (red), *S. cerevisae* YOL151W (gray).

A further insight and analogical comparison of the modeled structure of XP_445913.1 with other proteins exhibited that the conserved catalytic residues such as S134, Y175 and K179 were similar to the crystal structure of carbonyl reductase from *Sporobolomyces salmonicolor* and other yeast proteins (Fig. 2). In addition, similarities have been obtained for a lot of other amino acids too such as amino acids spanning hydrophobic channel of the 2 proteins, such as Phe 94→Phe 97, Trp 226→Val229, Pro241→Ala238, ILeu172→Leu174. However, for XP_445913.1, Q168 and E244 make the beginning of the channel little less hydrophobic than the crystal structure (1UJM) where there is Pro170 and Leu 241 in the equivalent positions (Table 1).

**Table 1.** Comparison of binding site.

	Catalytic residues			Hydrophobic channel					
*C. glabrata*	S133	Y 175	K 179	F 94	I 172	F 245	V229	P 241	—
*S. salmonicolor*	S134	Y 177	K 181	F 97	L 174	L 241	W 226	A 238	P170
*S. cerevisae*YDR541C	S129	Y 167	K 171	Y 89	I 164	P 235	—	—	—
*S. cerevisae*YGL157W	S131	Y 169	K 173	F 91	V 166	Y 239	I223	—	—
*S. cerevisae*YOL151W	S127	Y 165	K 169	—	V 162	I 232	—	—	P161

**Figure 3.**
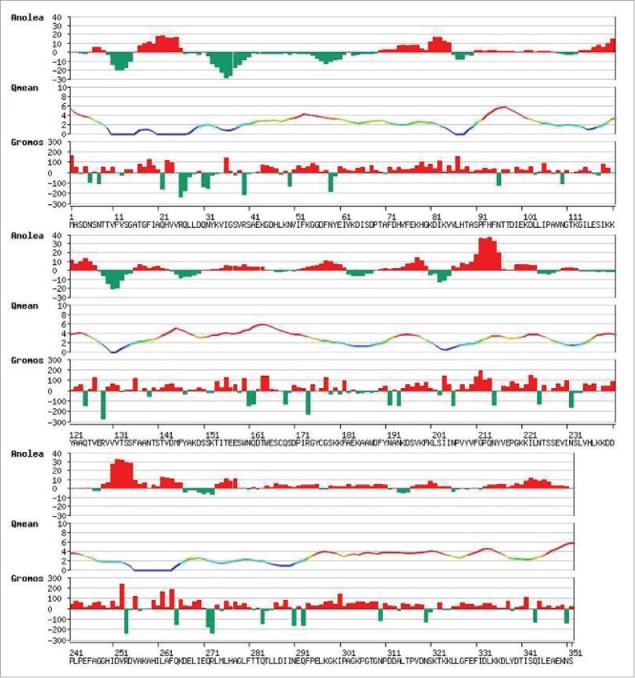
ANOLEA, QMEAN and GROMOS plot of modelled XP_445913.1 from *Candida glabrata* CBS138.

For estimation of modeling parameters, several parameters of our model has been performed such as ANOLEA (Atomic Non Local Environment),^12^ QMEAN (Qualitative Model Energy Analysis),^13^ GROMOS (Groningen Molecular Simulation Computer Simulation Package).^14^ While ANOLEA calculates knowledge based distance dependent mean force potential, QMEAN evaluates the quality of the model based on certain scoring function and GROMOS is the force field based on molecular simulation. PROCHECK^15^ has been used for Ramachandran Plot. The modeled protein quality check with ANOLEA, QMEAN and GROMOS have been provided in Figure 3. The model quality assessment has been performed using the Swiss-model workspace.^16^

**Figure 4.**
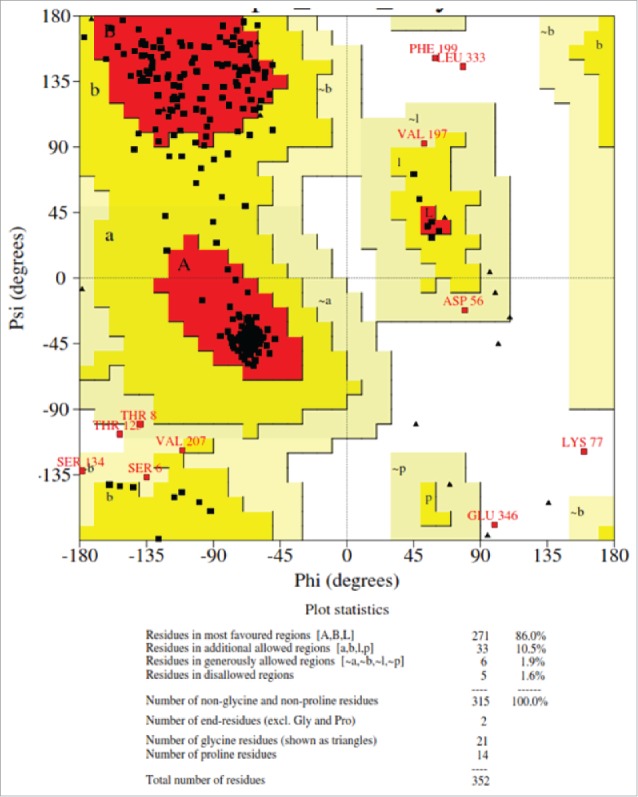
Ramachandran Plot of modelled XP_445913.1 from *Candida glabrata* CBS138.

**Figure 5.**
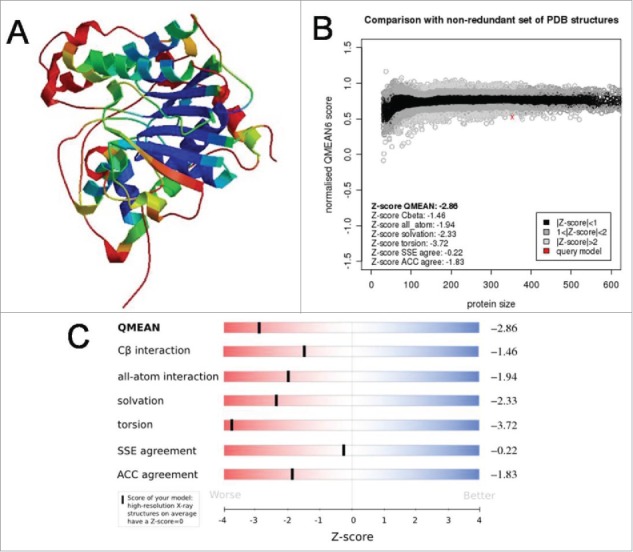
Different model validation parameters of modeleld XP_445913.1 from *Candida glabrata* CBS138. A. Coloring residue plot with errors (blue depicting most reliable residues while red suggests potentially unreliable residues). B. Psuedo-energy plot of contributing terms with their *z-scores*. C. QMEAN6 plot with query residues.

Ramachandran plot has been performed to assess the model quality by analyzing the favored, allowed and generously allowed perturbations of residue-residue interaction (Fig. 4). From the plot, it can be observed that most of the residues have been clustered in α and β regions with a very few outliers suggesting that most of the interactions are favored folding interactions. Total quality of the model is assessed based on the reliability model and was found to be QMEAN6 of 0.528.The coloring residues plot with respect to errors (Fig. 5A) and normalized QMEAN6 plot with respect to query protein residues (Fig. 5B) have been provided to demonstrate the CR model accuracy. The pseudo energy plot of the contributing terms (Fig. 5C) has also been provided with their *z-scores* (with respect to the scores obtained from high-resolution structures in this protein subset). The scores obtained from high-resolution structures solved by X-ray crystallography has been taken as baseline score.

### Docking studies

**Figure 6.**
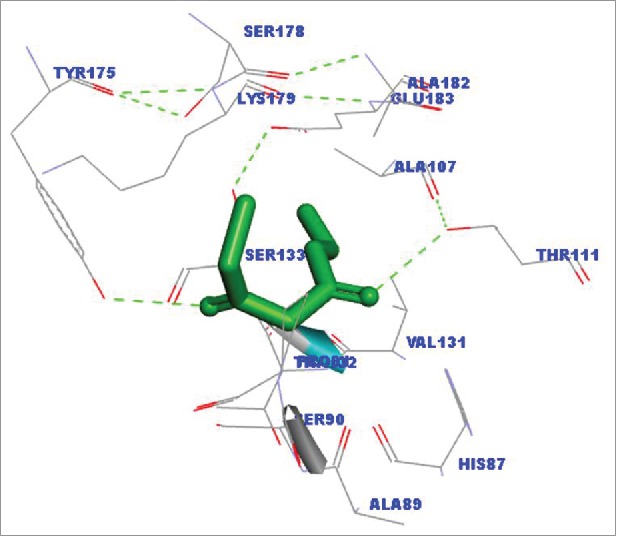
Binding site analysis of COBE in CR from *Candida glabrata* CBS138. The binding site analysis has been done using the standard protocol in Discovery Studio Visualizer 3.5. The dotted bond represents hydrogen bonding.

The docking studies were performed in AutoDockVina to evaluate the binding affinity of the substrate with the enzyme. The binding affinity of the target enzyme was compared with that of aforementioned standard AKR proteins to elucidate the enzyme potential as aldo-keto reductase against COBE. The energy minimized structure of COBE was prepared and converted to PDBQT format through MGL Tools 1.5.6. The modeled protein structures were freed from water molecules and inbound ligand by Discovery Studio 3.5 Visualizer. All the bonds and torsional angles of the ligand were allowed to rotate freely. *C. glabrata* CR was found to have more binding affinity (−4.6 Kcal/mol) compared with other same family of proteins (binding affinity ranging from −3.5 to −4.5 Kcal/mol) (Table 2). A binding site analysis displayed that the substrate can fit nicely into the hydrophobic cavity of the enzyme and the amino acids especially T111 and Y175 can form hydrogen bonds with the substrate carbonyl oxygen atom (Fig. 6).

**Table 2.** Docking scores of proteins.

Protein	Binding affinity (Kcal/mol)
*C. glabrata* XP_445913.1	−4.6
*S. cerevisiae* YDR541c	−3.9
*S. cerevisiae* YGL157w	−3.5
*S. cerevisiae* YGL157w	−4.5
AKR protein from *S. salmonicolor*	−3.7

**Figure 7.**
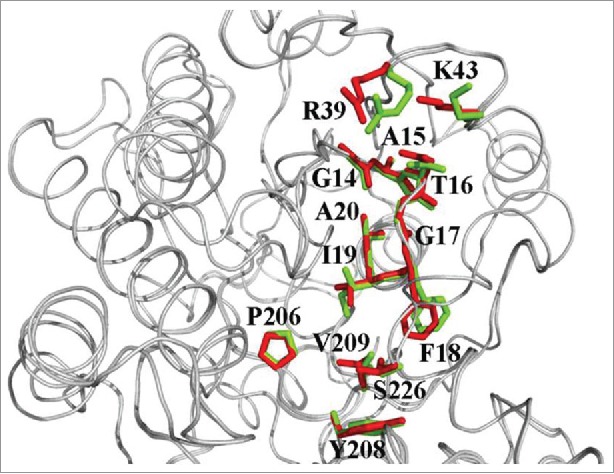
Positions of important amino acids for NADPH binding for *C. glabrata* (green) and *S. salmonicolor* (red).

NADPH dependence for *C. glabrata* CBS138 CR was interpreted by aligning the crystallographic structure of *S. salmonicolor* AKR protein together with that of *C. glabrata* and followed by comparing the cofactor attachment domain between the 2 proteins (Fig. 7). The cofactor domain mapping of the target protein together with comparing it with that of *S. salmonicolor* (1UJM) and *S. cerevisiae* (YDR541c, YGL157w and YOL151w) AKR proteins has been accomplished using standard alignment and labeling tools of PyMOL (www.pymol.org).

Thus homology model construction, alignment of important amino acids residues, analyzing substrate binding site, exploring cofactor domain and docking score comparisons with other standard ALR/ CR group of enzymes suggested that XP_445913.1 should belong to the same family of enzymes as the standards and thus we treated XP_445913.1 as CR (Carbonyl Reductase) group of protein from *Candida glabrata* CBS138. These findings encouraged us to try and explore its actual biocatalytic potential in realistic experimental condition. Thus we cloned the gene in heterologous vector, overexpressed and subsequently purified the protein, characterized through kinetic studies, calibrated through pH and temperature and finally exploited it in actual bioconversion through trinomial bioengineering as described in our earlier report.

### Optimization of bioconversion

**Figure 8.**
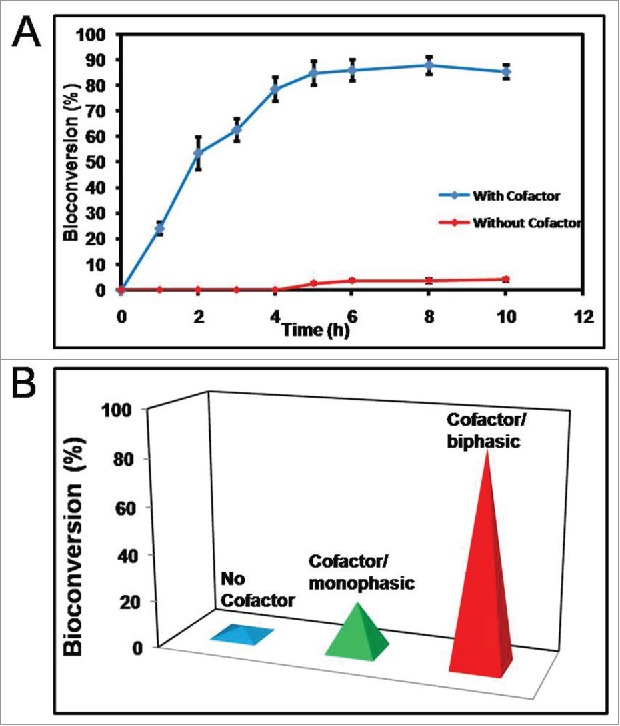
(*R*)-CHBE production profile in biotransformation reaction. A. Yield profile with or without cofactor regeneration B. Comparative study of bioconversion under 3 reaction schemes The data points are the average ( ± standard deviation) of 3 independent observations (n = 3).

The optimization of the reaction system was accomplished by analyzing the outputs with any 2 variables of the 3 key factors controlling the bioconversion such as monophasic system, biphasic system and NADPH as cofactor. Apparently, best yields were obtained when biphasic reaction system was used together with cofactor regeneration as reported by other researchers.^1-5^ As mentioned earlier,^10^ gene encoding Glucose dehydrogenase (GDH) from *Bacillus subtilis* has been cloned in the recombinant microbial cell factory together with CR from *Candida glabrata* CBS 138 to follow cofactor regeneration of NADPH from oxidized NADP^+^ *via* exogenously added substrate glucose. When whole cells over-expressing the CR and GDH proteins (cofactor regenerating) were used with previously reported conditions^10^ within biphasic reaction system, the substrate being in butyl acetate phase while recombinant host in the buffer phase, the product formation improved rapidly up to 4h and obtained steady-state within 6h (88.3% bioconversion from COBE to CHBE, Fig. 8A). In contrast, without cofactor regeneration even in biphasic system, only 5% bioconversion was achieved together with having a longer reaction time ∼8–10 h (Fig. 8A). However, a cofactor regenerating system within single buffer phase (0.1 M Potassium phosphate buffer, pH 7.5) produced a bioconversion of 20.80% necessitating the contribution of cofactor in enhancing the product yield. Figure 8B depicts a comparative portfolio of bioconversion under 3 aforementioned conditions.

### DNA shuffling- Library formation and mutant selection for future host construction

We used error-prone PCR (ep-PCR) in our previous study^10^ to construct highly stress tolerant mutant as our biocatalysis host and thus achieved significant improvement of product yield with elongation of cell fitness during the biphasic reaction. However we are still in search for further improvement of cell phenotype as we believe that cell fitness can be a key factor to tower the product yield during such bioreaction. Many such strategies are under investigation, among them DNA shuffling has emerged as a successful tool to improve cell phenotype in multitude of conditions.^17-20^

DNA shuffling was performed with a little modification of the procedure as described by Stemmer, 1994^21^ taking whole *crp* operons of 3 ep-PCR mutants (M1 ∼ M3) as templates. The acquisition of M1∼ M3 and their amino acid mutations have been already described in our previous publication.^10^ 4 µg of total template DNA was used for DNA shuffling. The total template DNA was digested by DNAse-I at 15°C for 3 mins and subsequently 50–200 bp DNA fragments had been recovered from gel electrophoresis for further process. Afterwards, DNA fragments were subjected to undergo PCR without primer and finally chimeric *crp* was recovered by amplification with forward and reverse *crp* primer, the sequences as discussed in our previous publication.

The DNA shuffling library was finally constructed by cloning the chimeric *crp* into pACYC Duet-I plasmid with *Kpn* I and *Bam* HI and subsequent introduction into *E. coli* DH5α as discussed earlier.^10^ The introduction of chimeric *crp*-pACYC Duet-I conjugate into *E. coli* DH5α through electroporation yielded a variant library in the order of 10^4^∼10^5^. DNA shuffling mutants were grown in LBGMg medium (Bacto tryptone 10 g/L, Yeast extract 1 g/L, NaCl 10 g/L, Glucose 2 g/L, MgSO_4_ 10 mM) and the winner was selected by subjecting the mutant library in 3 rounds of selection with 0.23∼0.25% (v/v) toluene. 50 mL High Density Polypropylene centrifuge tubes (BD Bioscienes, USA) with parafilm sealing have been used for culturing the variant with proper oxygenation (37°C, 200 rpm). Proper dilution of the culture from each round was plated onto LBGMg-agar plates to isolate the colonies. Selected clones from the third round were taken, the plasmid DNA was isolated by mini-preparation with QIAGEN plasmid isolation kit (Qiagen, USA). The shuffled mutations were verified by DNA sequencing and relevant plasmids were re-introduced into fresh *E. coli* DH5α background to create fresh variants. The fresh variants were challenged in LBGMg medium under 0.4% – 0.5% (v/v) toluene pressure to select the variant with best growth profile.

**Figure 9.**
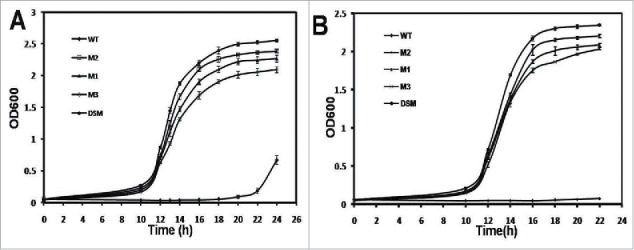
Tolerance profile of DNA shuffling mutant (DSM) with respect to parent mutants. The tolerance has been analyzed by growth of the mutant against hydrophobic solvent such as A. 0.40% (v/v) toluene B. 0.50% (v/v) toluene

One DNA shuffling mutant (DSM) was isolated and sequenced to reveal amino acid substitutions such as T127N F136I T208N. The DSM revealed better growth profile against higher concentrations of toluene (0.4% – 0.5% (v/v). Under 0.40% toluene, the DSM reached to OD_600_ ∼2.5 in 24 h where other mutants’ growth (M1∼ M3) remained within OD 2.0 ∼2.3 (Fig. 9A). In 0.50% toluene, where other reached a saturation OD ∼2.0 in 22 h, DSM reached a OD ∼2.4 in the same time (Fig. 9B).

## Discussion and conclusion

The CR from *Candida glabrata* was identified through BLASTP guided sequence search method. It is known that if there is a significant sequence match between 2 proteins, then it is highly likely that there are similarities in their functions. In this research work, it was found that there was a 55% ∼62% identity match between CR and 3 reported AKR family of proteins YDR151c, YGL157w and YOL151w.^11^ This suggests that the target protein might have an AKR like activity. The overlapping of active site between the target and the template proteins also imply similar tertiary structure of the proteins.

Since the CR has been structurally elusive together with YDR151c, YGL157w and YOL151w, all the proteins have been modeled to churn any similarities between their structures as well as the active sites. The models were first validated then the active site comparison revealed that similar hydrophobic channels spanned the target and template protein residues including structurally elucidated proteins 1UJM and 1Y1P. Thus it is a plausible assumption the substrate COBE may bind with the proteins in the same way, however the specificity and affinity may change depending upon the hydrophobicity of the channel. Furthermore, an *in silico* docking of COBE with the proteins revealed that COBE has even more binding affinity toward CR than other template proteins. This really encouraged us to try the CR from *Candida glabrata* in realistic bioconversion.

One critical bottleneck for enzymatic bioconversion is the hydrophilicity of the enzyme since enzymes usually are dissolved in buffered system for prolonging its stability and activity. Thus, a prior investigation of CR in this sort through GRAVY (grand average of hydropathicity) analysis (http://web.expasy.org/cgi-bin/protparam/protparam) revealed a negative gravy index as −0.359. This indicated protein's hydrophilicity^22^ thus advocating its competence in buffer mediated bioconversion. Then we challenged the enzyme in actual bioconversion and optimized it through whole cell biotransformation.

It is important to note that biphasic biotransformation provides better output in this reaction. The reason being that improved solubility of COBE in the organic phase (compared with aqueous) enhances the substrate carriage to the host cells. Also, COBE undergoes partial hydrolysis in aqueous phase which retards the rate of reaction in the monophasic biotransformation (data not shown). Cofactors are required to supply the hydrogen for the biotransformation.^1-5,23^

Harboring the aforementioned highly efficient recombinant enzyme for improved biotranformation, we have tried to overcome secondary challenges in biphasic biocatalysis such as tolerance of the host cell toward organic phase and improved substrate uptake inside the host cell reported in our previous publication.^10^ We have demonstrated that error prone PCR has been undertaken to improve cell phenotype during biocatalysis. Also, cell phenotype has been improved by DNA shuffling. For DNA shuffling we have used whole *crp* operon from the mutants because the *crp* operon contains 3 parts: the transcription factor binding site (Transcription Factor B involving 2 CRP-cAMP binding sites, 4 FISbinding sites, and 3 *crp* native promoters), the*crp* gene, and a specifically designed *rrn*B terminator.^24,25^ The resultant *in vitro* recombination yielded a super-performing mutant (DSM) which showed improved performance than the mothers against extremely hydrophobic toluene. Toluene has been selected as the challenging solvent because a “winner” against such extremely hydrophobic solvent would have good chance to survive other organic solvents too. LBGMg medium has been a choice of medium for survival of colonies against organic solvent.^26^ The DSM combines mutations at 3 regions namely T127 (in the cAMP binding pocket-cα helix that stabilizes cAMP- CRP binding),^27^ F136 (stabilizing interdomain hinge)^28^ and T208 (in the DNA binding domain).^27^ We propose that implementation of DNA Shuffling mutant in future biotransformation might improve the product titer even better and might gain other applications as well.
